# Pernicious Anemia Mimicking Thrombotic Thrombocytopenic Purpura in the Setting of Severe Vitamin B12 Deficiency

**DOI:** 10.7759/cureus.113023

**Published:** 2026-07-20

**Authors:** Constantine N Hrysikos, Hudson McKinney, Salvatore P Schaper, Mary McAtee

**Affiliations:** 1 Osteopathic Medicine, Edward Via College of Osteopathic Medicine, Spartanburg, USA; 2 Radiology, Spartanburg Regional Healthcare System, Spartanburg, USA; 3 Family Medicine, Spartanburg Regional Healthcare System, Spartanburg, USA

**Keywords:** adamts 13, pernicious anaemia, pseudo-thrombotic microangiopathy, thrombotic thrombocytopenic purpura, vitamin b 12 deficiency

## Abstract

Pernicious anemia (PA) is an autoimmune-mediated vitamin B12 deficiency that classically presents with macrocytic anemia and neurologic dysfunction. The case described highlights a rare variant of PA that paralleled concerns for microangiopathic hemolytic anemia (MAHA) like thrombotic thrombocytopenic purpura (TTP). A 40-year-old African American female with limited past medical history presented with progressive fatigue, lower-extremity weakness, gait instability, and ataxia, accompanied by chronic gastrointestinal symptoms, weight loss, and menorrhagia. Initial evaluation revealed profound macrocytosis, pancytopenia, laboratory evidence of hemolysis, including an elevated lactate dehydrogenase (LDH) and undetectable haptoglobin level without evidence of acute kidney injury (AKI), and neurologic deficits concerning for subacute combined degeneration. This case was complicated by right lower lobe pneumonia, urinary tract infection, and a left Bartholin cyst. Further testing demonstrated elevated methylmalonic acid and homocysteine levels, positive intrinsic factor (IF) antibodies, and only mildly reduced ADAMTS13 activity, which confirmed the diagnosis of PA. The patient was treated with parenteral vitamin B12, folate, and supportive care, resulting in gradual recovery. Although inpatient rehabilitation was recommended, the patient declined and was instead discharged home with arrangements for outpatient follow-up and lifelong intramuscular vitamin B12 replacement therapy. This case underscores the importance of recognizing that, in rare cases, PA can present as a mimicker of thrombotic microangiopathies such as TTP, as early identification can prevent unnecessary therapeutic intervention, reduce morbidity, and facilitate timely, disease-specific therapy.

## Introduction

Pernicious anemia (PA) is an autoimmune cause of macrocytic anemia due to vitamin B12 (cobalamin) deficiency from the loss of intrinsic factor (IF), a glycoprotein that aids in its absorption [1,2]. Autoantibodies against gastric parietal cell H⁺/K⁺-ATPase lead to IF deficiency, which impairs cobalamin absorption in the distal ileum [3,4]. Vitamin B12 deficiency disrupts DNA synthesis, causing megaloblastic hematopoiesis with ineffective erythropoiesis and intramedullary erythroid precursor destruction. This results in markedly elevated lactate dehydrogenase (LDH), inappropriately low reticulocyte responses despite severe anemia, and mild bilirubin elevation. Patients commonly present with fatigue, pallor, atrophic glossitis, and symmetric lower extremity weakness. Untreated PA may progress to subacute combined degeneration of the posterior and lateral spinal columns, manifesting as impaired vibration and proprioception, upper motor neuron signs (spasticity, hyperreflexia, extensor plantar responses), and ataxia. Less common features include psychiatric symptoms, urinary incontinence, dyspepsia, and visual or olfactory disturbances. 

PA can affect any age group, but the median age at diagnosis is 70-80 years old [4]. The disease is more common among those of African and European ancestry, with the highest prevalence in Northern Europeans. The mean age at presentation among black women, 53 +/- 16 years, is significantly lower than that of all the others [5]. PA has often been linked to other autoimmune conditions such as thyroid disease (40%), type 1 diabetes (10%), and vitiligo, suggesting that similar immunologic mechanisms either trigger or accelerate autoimmune gastritis [6]. Some environmental factors like tobacco use, a high-salt diet, and possibly chronic acid reflux can contribute to atrophic gastritis [7]. 

The diagnosis of PA is made by a combination of clinical manifestations, lab values, imaging, and pathology reports. The most common laboratory hematologic manifestations of PA include megaloblastic anemia with hyper-segmented neutrophils, elevated mean corpuscular volume (MCV) (>100 fL), and a low vitamin B12 level, which are the hallmark findings. IF antibodies, while highly specific for pernicious anemia, demonstrate a sensitivity of 60%, limiting their utility as a standalone diagnostic marker [8]. Black women demonstrate a higher prevalence of IF than the general population diagnosed with PA, with one systematic review demonstrating 96% of black women tested positive for circulating IF antibodies [5]. Another study found IF blocking antibodies in 85% of Black women, which contrasts with the usual prevalence in the general PA population [9]. When present, they are considered diagnostic, but the low sensitivity does not exclude PA from the diagnosis. Anti-parietal cells are found in 90%, making them highly sensitive, but they have low specificity as they are also seen in atrophic gastritis without megaloblastic anemia and in other autoimmune disorders [8]. Conversely, Black women under age 50 have a significantly lower prevalence of anti-parietal cell antibodies compared to European and Latin-American patients or even older Black women. Serum gastrin levels provide excellent diagnostic accuracy when stratified by proton pump inhibitor use. Mean serum gastrin levels in PA are markedly elevated (1,518 pg/mL) while values in individuals without PA range from 20-100 pg/mL. [3]. Methylmalonic acid (MMA) levels are elevated in >98% of patients with B12 deficiency, including those with only neurologic symptoms. Levels >500 nmol/L are suggestive of B12 deficiency, with 86% of patients with megaloblastic anemia having levels >1,000 nmol/L. While both B12 and folate (B9) deficiency can cause megaloblastic anemia and elevated homocysteine levels, MMA elevation is characteristically seen in B12 deficiency alone, making it a useful marker to differentiate between them [4]. An esophagogastroduodenoscopy (EGD) with biopsy provides definitive diagnosis for atrophic gastritis, histologically, though approximately 20% of patients are seronegative for antibodies [10]. 

In chronic, severe cases of PA, leukopenia, thrombocytopenia, and even pancytopenia can be appreciated. One rare manifestation is known as pseudo-thrombotic microangiopathy (pseudo-TMA) - a distinct disease process that can mimic TTP with schistocytes on blood smear, low haptoglobin due to hemolysis, thrombocytopenia, and an elevated LDH. Vitamin B12 serves as a cofactor for methionine synthase and L-methylmalonyl-CoA mutase, and its deficiency disrupts folate-dependent thymidylate synthesis, impairing the conversion of deoxyuridine monophosphate (dUMP) to deoxythymidine monophosphate (dTMP). Excess dTMP is misincorporated into DNA, and subsequent excision by DNA repair enzymes without available replacement nucleotides causes DNA strand breaks and fragmentation. This defective DNA synthesis produces structurally abnormal megaloblastic erythroid precursors that undergo premature apoptosis within the marrow (intramedullary hemolysis), releasing intracellular LDH and unconjugated bilirubin into the serum while consuming haptoglobin. The resulting laboratory profile - markedly elevated LDH, elevated indirect bilirubin, and undetectable haptoglobin - is biochemically indistinguishable from peripheral hemolytic anemia, including microangiopathic hemolytic anemia. Thrombocytopenia results from impaired megakaryocyte maturation due to the same DNA synthesis defect affecting all hematopoietic lineages. Megakaryocyte development is disrupted by megaloblastic changes, leading to reduced platelet production. Though the prevalence and rate of misdiagnosis of pseudo-TMA is unknown, a retrospective cohort study found that up to 6% of individuals with B12 deficiency presented with pseudo-TMA [11]. The misdiagnosis of pseudo-TMA can lead to unnecessary plasma exchange and a delay in appropriate treatment if not recognized. 

PA presenting as a microangiopathic hemolytic anemia can be distinguished from true TTP by several key laboratory features. A retrospective study showed that PA is associated with markedly elevated LDH levels (>2,500 U/L), reticulocytopenia with a reticulocyte count (13.1 x 10^9^) and a reticulocyte index < 2% [12]. The patients with a PA had preserved or mildly reduced platelet counts, profound neutropenia, and normal bilirubin levels. B12 deficiencies will also show elevated MMA levels, a positive IF antibody, and low cobalamin levels. In contrast, TTP typically demonstrates more modest LDH elevation (median 1,460 U/L), reticulocytosis (mean 265 x 10^9^), a reticulocyte index >2.5%, severe thrombocytopenia, normal neutrophil counts, and evidence of indirect hyperbilirubinemia [12]. TTP will also have decreased ADAMTS13 levels. 

## Case presentation

A 40-year-old African American female with a past medical history of menorrhagia and a left Bartholin cyst presented with progressive fatigue, bilateral lower-extremity weakness, and ataxia, which worsened over the previous week, impairing her ability to ambulate independently. Her symptoms were present intermittently for several years and were accompanied by chronic nausea, episodic bilious vomiting, constipation, unintentional weight loss, and menorrhagia. More recently, she developed a subacute cough and shortness of breath with exertion. She had limited follow-up with any medical provider for the previous 10 years. On arrival, the patient was hemodynamically unstable with tachycardia, hypotension, and lethargy. Physical examination revealed mild scleral icterus, lower extremity weakness, impaired sensation and proprioception below the knees, and gait ataxia. 

Laboratory evaluation revealed severe anemia (Hgb 3.2 g/dL) with macrocytosis (MCV 100.8 fL), elevated red cell distribution width (RDW) of >40%, thrombocytopenia (81x10³/uL), and leukopenia (3.6x10³/uL). Lactic acid was normal (1.0 mmol/L), but her procalcitonin was elevated (0.50 ng/mL). Other lab markers demonstrated intravascular hemolysis, including an elevated lactate dehydrogenase (LDH) (3250 U/L), undetectable haptoglobin (<30 mg/dL), and indirect hyperbilirubinemia (3.10 mg/dL). Her vitamin B12 level was undetectable (<50 pg/mL), and her vitamin D level was low (<4.0 ng/mL). Her iron and folate studies were normal. Reticulocyte count and percentage were elevated at 0.172x10⁶/uL and 6.34%, respectively, after the patient received B12 supplementation, indicating an appropriate marrow response when B12 was present. Renal function tests were negative for an acute kidney injury (AKI). Urinalysis was positive for protein (20 mg/dL), blood (1+), and white blood cells (36 hpf), indicative of acute bacterial cystitis; cultures returned positive for Pseudomonas aeruginosa and Klebsiella variicola. Atypical bacterial tests like Mycoplasma spp. were negative. Other pertinent positives of the imaging included a left-sided Bartholin cyst, a 4.5 cm right adnexal mass, and cholelithiasis without evidence of cholecystitis. 

Chest x-rays were unremarkable for an acute cardiopulmonary process. However, a computerized tomography (CT) abdomen/pelvis revealed opacities of the lower lobe of the right lung, suggesting a unilateral lower-lobe pneumonia. The magnetic resonance imaging (MRI) brain revealed the incidental finding of a 13 mm pineal cyst but was otherwise normal. The MRI cervical spine showed circumferential disc bulging from C4-T1 as well as mild canal stenosis and facet joint arthropathy from C4-C7 without foraminal narrowing. The MRI thoracic spine showed normal spinal cord morphology, no evidence of degenerative vertebral changes, foraminal narrowing, or canal stenosis. The MRI lumbar spine showed no acute abnormalities and no evidence of degenerative changes. 

The combination of severe anemia, thrombocytopenia, elevated LDH, and hemolysis raised initial concern for a hemolytic process. The differential diagnosis included thrombotic thrombocytopenic purpura (TTP), G6PD deficiency, warm or cold autoimmune hemolytic anemia, paroxysmal nocturnal hemoglobinuria, and immune thrombocytopenic purpura. Her vitamin B12 deficiency raised concern for PA or another malabsorptive disorder like celiac disease. 

The patient was admitted for hemodynamic instability, management of pancytopenia, hemolytic anemia, right lower lobe pneumonia, bacterial cystitis, and left Bartholin cyst. While in the ED, she was transfused with three units of packed red blood cells -- intramuscular vitamin B12 replacement was initiated promptly, along with vitamin D, folate, and thiamine supplementation. Due to cross-susceptibility, her pneumonia and urinary tract infection (UTI) were treated with intravenous (IV) cefepime, while the cyst was managed with oral trimethoprim-sulfamethoxazole. Drainage of the cyst was deferred because of persistent thrombocytopenia. 

Hematology/Oncology was consulted for evaluation, and they attributed the pancytopenia and hemolysis to a sequela of severe vitamin B12 deficiency due to an autoimmune process. Despite laboratory features concerning for thrombotic microangiopathy, plasma exchange was deferred given early identification of profound vitamin B12 deficiency and lack of clinical features consistent with TTP, including preserved mental status and absence of acute renal failure. 

Neurology was consulted for evaluation of progressive weakness and gait instability. MRI of the brain and spinal cord with and without contrast showed no evidence of subacute combined degeneration or acute myelopathy. The presence of peripheral neuropathy and mild ataxia was attributed to her chronic vitamin B12 deficiency. Gastroenterology evaluation, including upper endoscopy and duodenal biopsy, revealed no evidence of bleeding, malabsorption, or malignancy. Biopsies of the stomach & duodenum were obtained to rule out H. pylori and celiac disease, which were both negative. Obstetrics and Gynecology (OBGYN) was consulted for her Bartholin cyst and gave recommendations regarding inpatient treatment and outpatient follow-up. 

Upon further evaluation, Direct antiglobulin testing (DAT) was positive for complement (C3), raising concern for immune-mediated hemolysis. The DAT IgG was negative. ADAMTS13 activity was mildly reduced (65%) but not consistent with TTP. Peripheral smear evaluation did not demonstrate significant schistocytosis. Subsequent testing confirmed positive IF antibodies (72.6 AU/mL), elevated methylmalonic acid (MMA) (538 nmol/L), and elevated homocysteine (25.1 umol/L), confirming PA as the underlying etiology. 

Over the course of admission, the patient demonstrated gradual recovery, with adequate nutrition, improvement of pancytopenia, and replenishment of vitamin B12 and folate. Her neurologic symptoms and functional status improved with treatment. Physical and occupational therapy evaluations recommended transfer to an acute inpatient rehabilitation facility, which the patient declined. At discharge, she was prescribed lifelong intramuscular vitamin B12 therapy, along with vitamin D and folate supplementation, and was counseled extensively on the chronic nature of PA and the risk of recurrence with nonadherence. She was also given oral antibiotics to finish the treatment course for her pneumonia, UTI, and Bartholin cyst. Refer to Table 1 for lab value reference ranges and interpretations.

**Table 1:** Patient Labs Values, Reference Ranges, and Interpretations

Laboratory Test	Patient Value	Reference Range	Interpretation
Hematology			
Hemoglobin (Hgb)	3.2 g/dL (grams per deciliter)	12.0–16.0 g/dL (female)	Critically low
Mean Corpuscular Volume (MCV)	100.8 fL (femtoliters)	80–100 fL	Elevated (macrocytic)
Red Cell Distribution Width (RDW)	>40%	11.5–14.5%	Markedly elevated
Platelet Count	81 × 10³/µL (thousands per microliter)	150–400 × 10³/µL	Low (thrombocytopenia)
White Blood Cell Count (WBC)	3.6 × 10³/µL	4.5–11.0 × 10³/µL	Low (leukopenia)
Reticulocyte Count	0.172 × 10⁶/µL (millions per microliter)	0.020–0.100 × 10⁶/µL	Elevated (post-B12 repletion)
Reticulocyte Percentage	6.34%	0.5–2.5%	Elevated (post-B12 repletion)
Peripheral Smear – Schistocytes	Not significant	None	Normal
Hemolysis Markers			
Lactate Dehydrogenase (LDH)	3,250 U/L (units per liter)	140–280 U/L	Markedly elevated
Haptoglobin	<30 mg/dL (milligrams per deciliter)	30–200 mg/dL	Undetectable
Indirect Bilirubin	3.10 mg/dL	0.1–1.0 mg/dL	Elevated
Direct Antiglobulin Test (DAT) – C3	Positive	Negative	Abnormal
Direct Antiglobulin Test (DAT) – IgG	Negative	Negative	Normal
ADAMTS13 Activity	65%	>67% (normal); 10% suggests thrombotic thrombocytopenic purpura (TTP)	Mildly reduced
Nutritional / Metabolic			
Vitamin B12	50 pg/mL (picograms per milliliter)	200–900 pg/mL	Undetectable
Vitamin D	4.0 ng/mL (nanograms per milliliter)	30–100 ng/mL	Critically low
Folate	Normal	2.7–17.0 ng/mL	Normal
Iron Studies	Normal	Varies by parameter	Normal
Methylmalonic Acid (MMA)	538 nmol/L (nanomoles per liter)	87–318 nmol/L	Elevated
Homocysteine	25.1 µmol/L (micromoles per liter)	5–15 µmol/L	Elevated
Autoimmune / Serologic			
Intrinsic Factor (IF) Antibodies	72.6 AU/mL (arbitrary units per milliliter)	1.53 AU/mL (negative)	Positive (confirms PA)
Inflammatory / Infectious			
Lactic Acid	1.0 mmol/L (millimoles per liter)	0.5–2.0 mmol/L	Normal
Procalcitonin	0.50 ng/mL (nanograms per deciliter)	0.10 ng/mL	Elevated
Mycoplasma spp.	Negative	Negative	Normal
Urinalysis			
Protein	20 mg/dL	Negative	Abnormal
Blood	1	Negative	Abnormal
White Blood Cells	36 HPF (high-power field)	0–5 HPF	Elevated
Urine Culture			
Organisms	Pseudomonas aeruginosa, Klebsiella variicola	No growth	Positive (polymicrobial urinary tract infection)

## Discussion

Multiple case reports have documented patients with PA who were initially misdiagnosed with TTP and treated with plasma exchange before the correct diagnosis was established [13-15].  The pathophysiology underlying pseudo-TMA involves homocysteine-mediated endothelial dysfunction, leading to microvascular thrombus formation, combined with ineffective erythropoiesis, causing significant red cell fragmentation. Given these overlapping features, a recent case review proposed that acquired vitamin B12 deficiency should be listed as a separate entity within the classification of thrombotic microangiopathies [16].  

The prevalence of PA increases in older populations - estimated at 2-3% in those older than 65 years compared to 0.1% in the general population. Across all racial populations, the median age at diagnosis is approximately 60 years old, while black women present at a younger mean age of 52±16 years. Notably, certain studies showed that 29.6% of black patients were <40 years old at diagnosis, and 14 % were 30 years old [9]. Although PA most commonly affects older adults of Northern European ancestry, this patient’s presentation illustrates that severe disease can occur earlier in life and across diverse populations, particularly black women. 

Differences in antibody prevalence patterns across patient populations have important implications for the diagnosis of PA. In Black women, IF antibodies occur at substantially higher rates than in the general PA population, while anti-parietal cell antibodies-particularly in those under 50 years of age-are less frequently detected [17]. This atypical antibody distribution may lead to underdiagnosis if testing relies predominantly on anti-parietal cell antibodies or age-based pretest assumptions. Elevated MMA levels in those with B12 deficiency may make it the more pertinent lab value for confirming the presence or absence of B12 deficiency. Additionally, the presence or absence of the IF antibodies and/or anti-parietal antibodies does not definitively exclude PA as the primary etiology given the low sensitivity and low specificity of these two tests, respectively.  

Several laboratory features help distinguish pseudo-TMA from true TTP. Vitamin B12 deficiency is characterized by markedly elevated LDH-often exceeding levels seen in TTP-along with reticulocytopenia, neutropenia, relatively preserved platelet counts, and normal or mildly elevated bilirubin, reflecting ineffective erythropoiesis rather than peripheral hemolysis. In contrast, TTP typically presents with severe thrombocytopenia, reticulocytosis, and indirect hyperbilirubinemia. Reticulocyte indices can help differentiate the two pathologies: patients with PA have low reticulocyte counts and indices (<2%), whereas TTP demonstrates appropriately elevated reticulocytosis (>2.5%) [12]. In this case, rapid reticulocyte recovery following B12 supplementation supported the diagnosis. A retrospective study comparing pseudo-TMA and TTP confirmed these distinctions, showing significantly higher LDH (7310 vs. 1460 IU/L, p=0.01), higher platelet counts (73 vs. 12.5 × 10⁹/L, p=0.0023), and lower reticulocyte (13.1 vs. 265.5 × 10⁹/L, p=0.0012) and neutrophil counts (1.3 vs. 5.1 × 10⁹/L, p=0.0023) in pseudo-TMA. Diagnosis may be confounded by falsely normal B12 levels due to IF antibody interference, masked macrocytosis from schistocytosis, and elevated platelets, hemolysis, absence of active cancer, history of solid organ or stem cell transplant, mean corpuscular volume, international normalized ratio, and creatinine (PLASMIC) scores [13]. Clinicians should maintain a high index of suspicion of B12 deficiency in patients presenting with apparent microangiopathic hemolytic anemia (MAHA), very high LDH (>2500 U/L), low reticulocyte counts, elevated MCV, and near-normal bilirubin, as prompt vitamin replacement leads to complete hematologic recovery. 

Thrombotic microangiopathies like TTP present with complement abnormalities and positive autoimmune titers. The patient tested positive for anti-C3 Coombs and negative DAT IgG, which suggests the presence of IgM-mediated hemolytic processes like cold-agglutinin disease or systemic lupus erythematosus (SLE). However, positive DAT binding to C3 fragments on RBCs is a known abnormality seen in approximately 31% of patients with megaloblastic anemia [18]. C3 can be significantly reduced in B12 deficiency, and the reduced levels correlate with the degree of anemia [19]. C3 bound to RBCs rather than IgG likely reflects the production of structurally abnormal RBCs due to vitamin deficiency rather than an immune-mediated attack. Both serum C3 abnormalities and DAT positivity are reversible in PA with B12 deficiency. In retrospect, follow-up labs such as a cold agglutinin titer and repeat anti-C3 should have been ordered to confirm the absence of cold agglutinin disease.

Reduced ADAMTS13 activity is a hallmark finding in TTP that is not found in the PA disease process. In this case, the patient had a mildly reduced ADAMTS13 activity, but the degree of reduction did not meet diagnostic criteria. TTP is diagnosed when ADAMTS13 activity is <10% of normal (>66.8%), and most patients with acute TTP have an activity level of 5% [20,21]. While it does not completely exclude the diagnosis, the patient’s measured ADAMTS13 level (65%) was far greater than 10% of normal, making TTP far less likely to be the diagnosis. 

The PLASMIC score is a seven-component clinical prediction tool used to estimate the probability of severe ADAMTS13 deficiency in patients presenting with thrombotic microangiopathy. The scores are stratified as low (0-4), intermediate (5), or high risk (6-7) for TTP. A systematic review and meta-analysis of 13 validation cohorts demonstrated that a PLASMIC score ≥5 carries a sensitivity of 99% and negative predictive value of 99% at a TTP prevalence of 35%, making it useful to rule out TTP; however, its specificity at this threshold is only 57%, meaning a substantial proportion of patients scoring ≥5 will not have TTP and may be unnecessarily treated with empiric plasma exchange [22]. The patient's PLASMIC score of 5 met criteria for hemolysis variables (indirect bilirubin 3.10 mg/dL, undetectable haptoglobin, reticulocyte percentage 6.34%), but she failed to meet criteria for other variables for scoring (active malignancy, no transplant history, and preserved renal function, for thrombocytopenia <30 × 10⁹/L or MCV <90 fL). Notably, the patient's age of 40 years placed her at the threshold of reported decline in score sensitivity, where detection rates fall from 91.4% in patients aged 18-39 to 78.3% in those aged 40-59 [23]. Despite an intermediate score that would prompt consideration of empiric therapeutic plasma exchange, the diagnosis of TTP was further made less likely by an ADAMTS13 activity of 65% - well above the <10% threshold required for TTP - and the absence of significant schistocytosis on peripheral smear. This case illustrates a key limitation of the PLASMIC score: its components are not entirely specific to ADAMTS13 deficiency, and an intermediate score in the setting of profound nutritional deficiency requires context-driven interpretation prior to starting empiric plasma exchange. Nevertheless, the PLASMIC score remains a validated clinical prediction tool in hematology, with external validation demonstrating a c-statistic of 0.94, and its utility in rapidly triaging undifferentiated TMA patients toward or away from empiric plasma exchange is well-established in populations without confounding nutritional or erythropoietic pathology.

AKI is not a recognized feature of PA, whereas it occurs in approximately 48-59% of patients with TTP, though cases are typically mild. In one case series, 58.7% of TTP patients presented with AKI, including 46.3% with stage 3 AKI, and 25.9% required renal replacement therapy [24]. In this case, the patient had evidence of an acute UTI on urinalysis, but her normal serum creatinine (0.94) and normal urine output made the presence of an acute AKI unlikely. 

Spinal cord MRI demonstrates highly variable sensitivity for detecting T2 hyperintensity in SCD, with the largest study by Jain et al. finding that only 14.8% of patients with biochemically confirmed B12 deficiency presenting with clinical features demonstrated cord abnormalities on MRI. This variability likely reflects differences in disease stage, field strength, and patient selection rather than true diagnostic heterogeneity [25]. When present, T2 signal abnormalities follow characteristic axial patterns -- the "inverted V sign" in the cervical cord and the "binocular" or "dumbbell" sign in the thoracic cord -- that are highly specific and, when identified, strongly support the diagnosis. The complete resolution of cord signal abnormalities following B12 repletion underscores the reversible nature of the underlying vacuolar demyelination and reinforces the value of treatment response as a diagnostic criterion. Lastly, clinicians should remain alert to the inverse relationship between hematologic and neurologic severity first described by Stabler, whereby patients presenting with severe myelopathy may present with minimal or absent megaloblastic changes, further emphasizing that the diagnosis of SCD must rest on clinical suspicion and biochemical confirmation rather than imaging or hematologic findings alone [4].

In this case, early identification of profound vitamin B12 deficiency, absence of renal failure or encephalopathy, and lack of significant schistocytosis supported a diagnosis of PA. Although ADAMTS13 activity was mildly reduced, it was not in the range typically seen in TTP, further reinforcing an alternative diagnosis. Prompt initiation of parenteral vitamin B12 led to hematologic recovery and neurologic improvement, underscoring the reversibility of this condition when appropriately treated. 

## Conclusions

PA should be recognized as a rare but important mimicker of TTP. Severe vitamin B12 deficiency can present with pancytopenia, hemolysis, and striking LDH elevation, closely resembling thrombotic microangiopathy. Careful evaluation of reticulocyte response, bilirubin levels, neutrophil counts, renal function, and ADAMTS13 activity can help distinguish pseudo-TMA from true TTP. Early identification of this entity is essential to avoid unnecessary plasma exchange and to initiate timely vitamin B12 replacement, which can result in rapid hematologic recovery and improvement of neurologic symptoms. This case highlights the importance of maintaining a broad differential diagnosis when evaluating suspected thrombotic microangiopathy, particularly in patients with macrocytosis or neurologic findings. 

## References

[1] Yocum AD, Patel M, Palocko B, Simon EL (2023). Primary neurologic symptoms: have you considered pernicious anemia?. J Emerg Med.

[2] Esposito G, Dottori L, Pivetta G, Ligato I, Dilaghi E, Lahner E (2022). Pernicious anemia: the hematological presentation of a multifaceted disorder caused by cobalamin deficiency. Nutrients.

[3] Toh BH (2017). Pathophysiology and laboratory diagnosis of pernicious anemia. Immunol Res.

[4] Stabler SP (2013). Vitamin B12 Deficiency. New England Journal of Medicine.

[5] Carmel R, Johnson CS (1978). Racial patterns in pernicious anemia. Early age at onset and increased frequency of intrinsic-factor antibody in black women. N Engl J Med.

[6] Lahner E, Annibale B (2009). Pernicious anemia: new insights from a gastroenterological point of view. World J Gastroenterol.

[7] Shah SC, Piazuelo MB, Kuipers EJ, Li D (2021). AGA clinical practice update on the diagnosis and management of atrophic gastritis: expert review. Gastroenterology.

[8] Bizzaro N, Antico A (2014). Diagnosis and classification of pernicious anemia. Autoimmun Rev.

[9] Solanki DL, Jacobson RJ, Green R, McKibbon J, Berdoff R (1981). Pernicious anemia in blacks. A study of 64 patients from Washington, D. C., and Johannesburg, South Africa. Am J Clin Pathol.

[10] Patel H, McGuirk R (2025). Vitamin B12 Deficiency: common questions and answers. Am Fam Physician.

[11] Koshy AG, Freed JA (2020). Clinical features of vitamin B12 deficiency mimicking thrombotic microangiopathy. Br J Haematol.

[12] Noël N, Maigné G, Tertian G (2013). Hemolysis and schistocytosis in the emergency department: consider pseudothrombotic microangiopathy related to vitamin B12 deficiency. QJM.

[13] Mohammad Z, Ananthaneni A, Fontenot A, Ramadas P, Nour Salloum M (2024). Unusual case of pernicious anaemia masquerading as thrombotic thrombocytopenic purpura in the setting of multiple normal vitamin B12 deficiency parameters: preventing anchoring and overdiagnosis. Fam Pract.

[14] Malla M, Seetharam M (2016). To treat or not to treat: a rare case of pseudo-thrombotic thrombocytopenic purpura in a Jehovah's Witness. Transfusion.

[15] Green R (2012). Anemias beyond B12 and iron deficiency: the buzz about other B's, elementary, and nonelementary problems. Hematology Am Soc Hematol Educ Program.

[16] Lämmle B, Laemmle A (2024). Vitamin B12 deficiency misdiagnosed as TTP: What can we learn from it?. Br J Haematol.

[17] Carmel R, Ozturk G, Johnson CS, Tung KS, Terasaki PI (1981). Profiles of black and Latin-American patients having pernicious anemia. HLA antigens, lymphocytotoxic antibody, anti-parietal cell antibody serum gastrin levels, and ABO blood groups. Am J Clin Pathol.

[18] FO J, HA L (1965). The direct antiglobulin (Coombs) test in megaloblastic anaemia. J Clin Pathol.

[19] Reynolds E (2006). Vitamin B12, folic acid, and the nervous system. Lancet Neurol.

[20] Joly BS, Coppo P, Veyradier A (2017). Thrombotic thrombocytopenic purpura. Blood.

[21] Bianchi V, Robles R, Alberio L, Furlan M, Lämmle B (2002). Von Willebrand factor-cleaving protease (ADAMTS13) in thrombocytopenic disorders: a severely deficient activity is specific for thrombotic thrombocytopenic purpura. Blood.

[22] Paydary K, Banwell E, Tong J, Chen Y, Cuker A (2020). Diagnostic accuracy of the PLASMIC score in patients with suspected thrombotic thrombocytopenic purpura: a systematic review and meta-analysis. Transfusion.

[23] Pishko AM, Li A, Cuker A (2025). Immune thrombotic thrombocytopenic purpura: a review. JAMA.

[24] Zafrani L, Mariotte E, Darmon M (2015). Acute renal failure is prevalent in patients with thrombotic thrombocytopenic purpura associated with low plasma ADAMTS13 activity. J Thromb Haemost.

[25] Jain KK, Malhotra HS, Garg RK, Gupta PK, Roy B, Gupta RK (2014). Prevalence of MR imaging abnormalities in vitamin B12 deficiency patients presenting with clinical features of subacute combined degeneration of the spinal cord. J Neurol Sci.

